# The obliterated ureteric orifice: a nightmare for the pelvic surgeon or a routine-job for the endo-urologist? Case report of a standardized endoscopic approach

**DOI:** 10.1093/jscr/rjab642

**Published:** 2022-01-26

**Authors:** Sahana S Balakrishnan, Mithun M Kailavasan, Aristeidis A Alevizopoulos

## Abstract

We present the endoscopic management of two cases of complete ureteric occlusion at vesico-ureteral junction (VUJ) level following iatrogenic injury. Case 1 is a 60-year-old man who developed bilateral ureteric injury at the level of the VUJ following robot-assisted radical prostatectomy (RARP) for Gleason 3 + 4 = 7 T2bN0 prostate cancer. Case 2 is an 81-year-old man with history of recurrent G2pTa transitional cell carcinoma of the bladder originally diagnosed in 2005 and history of radical radiotherapy for prostate cancer. At his most recent transurethral resection of bladder tumour, the left ureteric orifice was not visualized. We describe step-by-step our technique in restoring continuity of the ureter with minimally invasive endoscopic approach, resulting in excellent long-term upper tract drainage for our patients. To our knowledge, combined utilization of a Collins knife to incise the area around the ureteric orifice to unearth them is not reported. We aim to report our technique and its outcomes.

## INTRODUCTION

Ureteric injuries are rare, most commonly secondary to iatrogenic injury with a reported rate of up to 0.3% following radical prostatectomy (RARP) [1]. Risk factors for iatrogenic ureteric injuries include conditions that alter normal anatomy such as advanced malignancy, prior surgery or irradiation and haemorrhage [2]. In this case series, we report step-by-step our technique in restoring continuity of the ureter to successfully manage a complete ureteric obstruction at the vesico-ureteric junction (VUJ).

## CASE 1

A 60-year-old gentleman with a presenting prostate-specific antigen (PSA) of 7.8 and transrectal biopsy-proven Gleason 3 + 4 = 7 (Grade 2) prostate adenocarcinoma underwent robot-assisted RARP for his malignancy. The pre-biopsy magnetic resonance imaging (MRI) reported a prostate of 44 cc in size with bilateral PI-RADS 4 lesions querying T3a staging on the right. During the RARP, the anastomosis step was more challenging than expected, with suboptimal views due to ongoing bleeding. Total blood loss was 500 ml.

In the immediate post-operative phase, the patient developed flank pain and became anuric. Non-contrast computed tomography (CT) revealed bilateral ureteric obstruction and extensive bilateral perinephric stranding with retroperitoneal fluid pronounced on the right, tracking anterior to the iliopsoas muscle. On Day 2 post-operatively, the patient underwent bilateral nephrostomies to relieve the obstruction. His creatinine receded from 320 mmol/L to <100 mmol/L within hours.

**Figure 1:**
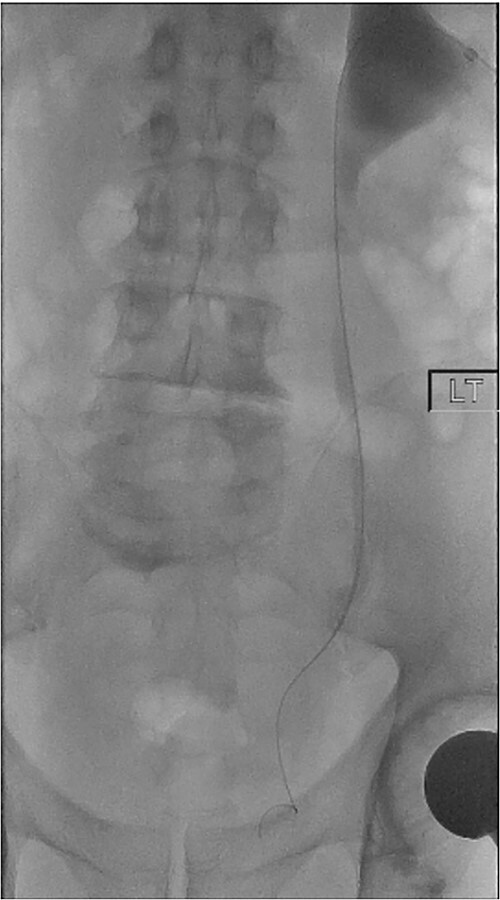
Antegrade left nephrostogram demonstrating left VUJ hold up.

On Day 5 post-operatively, bilateral nephrostograms revealed bilateral ureteric occlusion at the level of the VUJ with extravasation at the right side (Fig. 1). An attempted antegrade ureteric stent insertion failed. The patient was thereafter booked electively for a rigid cystoscopy and bilateral ureteric stenting 3 weeks following the original operation.

## CASE 2

This is a case of an 81-year-old man with history of multiple low-grade recurrences of G2pTa TCC of the bladder, initially diagnosed in 2005. He also had radical radiotherapy for intermediate risk prostate cancer. In April 2021, he underwent a transurethral resection of bladder tumour (TURBT) recurrence. The left ureteric orifice could not be identified, due to complete occlusion by scar tissue from previous TURBTs. His creatinine was stable at 125 mmol/L. A subsequent CT Urogram demonstrated new onset gross hydronephrosis with obstruction at level of VUJ. No contrast was detected within the left collecting system. A left nephrostomy was placed and attempted left antegrade stent followed, without success. MRI Pelvis with urographic phase demonstrated a left bladder wall recurrence at the area of the left VUJ and raised possibility of tumour presence at the left ureteric cul-de-sac.

The patient underwent elective cystoscopy, incision of the left ureteric orifice region, left rigid ureteroscopy with biopsies and laser ablation of distal ureteric tumour with insertion of stent.

## PROCEDURE

**Figure 2:**
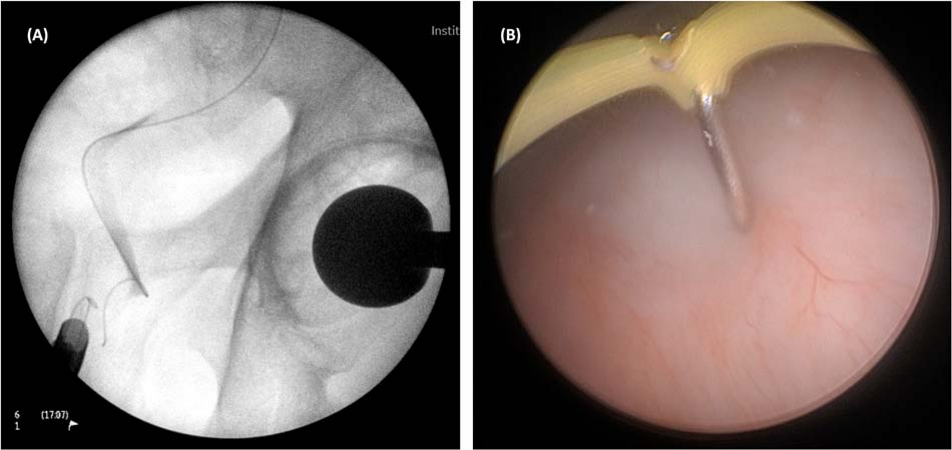
A combined visual- and x-ray-guided approach verified the exact location of the course of the intravesical intramural right ureter, in correlation to the resectoscope. (**A**) X-ray, (**B**) cystoscopic view.

**Figure 3:**
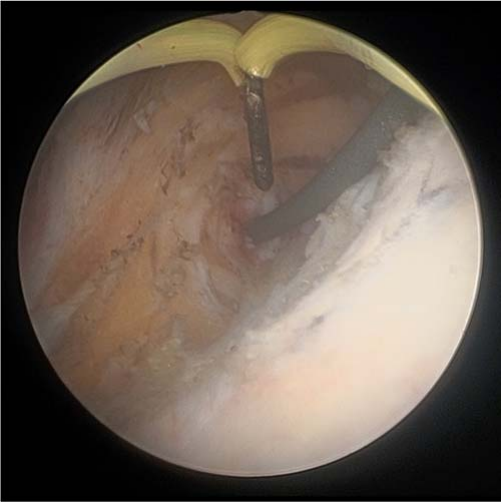
Cystoscopic view of exposed left VUJ with identification of terumo guidewire passed antegradely.

On both cases, under general or regional anaesthesia, a cystoscopy was performed and the interureteric bar was identified. A hybrid ‘sensor’ or ‘Terumo’ hydrophilic guidewire was advanced concurrently using the access from the existing nephrostomy tube with fluoroscopic assistance down to the level of the ureteric cul-de-sac. A combined visual- and x-ray-guided approach verified the exact location of the course of the intramural ureter, in correlation to the resectoscope and a Collins knife was used to incise the bladder mucosa (Fig. 2) above the level of the guidewire, on the virtual course of the intramural ureter exposing the intramural ureter. In all three attempts, the incision of bladder mucosa above the antegrade ureteric guidewire ended up with a successful identification of the guidewire, allowing access to the ureteric cul-de-sac. Stent graspers were used to snare the guidewire into the bladder (Fig. 3). With safety wire in place, management of the neo-orifice could then be performed.

**Figure 4:**
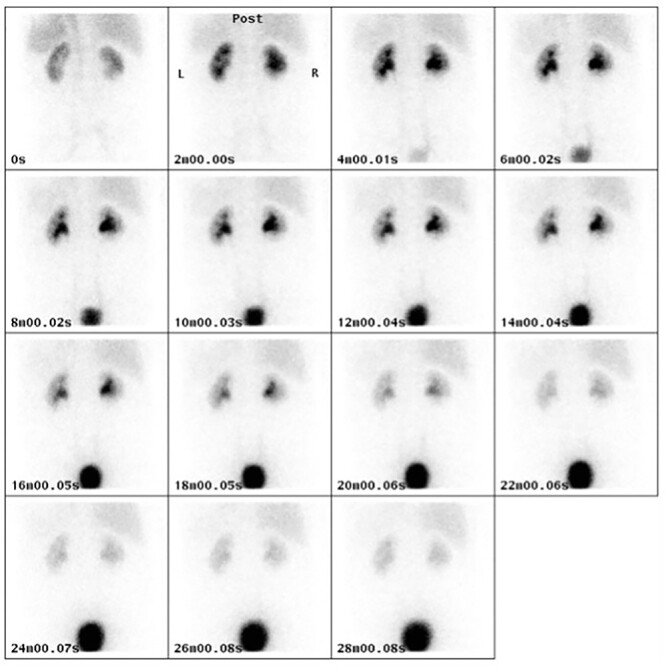
MAG3 renogram at 4 months post-operatively demonstrating normal uptake and excretion pattern in both kidneys.

In Case 1, balloon dilatation of the neo-orifices was followed by a successful retrograde ureteric stent insertion**.** Both nephrostomies were removed peri-operatively. Both ureteric stents were removed at ~2 months after the procedure using a flexible cystoscope. A follow-up MAG3 renogram at 4 months post-operatively (Fig. 4) revealed 47% and 53% function of right and left kidney, respectively. A further MAG3 renogram at 2 years post-operatively reported 48% and 52% function of right and left kidney, respectively with no evidence of hydronephrosis or obstruction. His current eGFR almost 3 years from the stent removal is 66 ml/min. It is thought that part of the patient’s renal function was lost due to the acute bilateral obstruction, but also due to fungal urosepsis following his endoscopic repair. This was managed successfully with prolonged oral Fluconazole, until the date of his stent removal.

In Case 2, a left ureteroscopy was performed over a second guidewire inserted retrogradely. This revealed a short segment (<2 cm) of superficial papillary tumour at the distal ureter, without mucosal abnormalities in the middle or proximal ureter. A 1.9Fr tipless basket was used to obtain histological tissue for analysis and a Holmium:YAG laser applied at 1 J × 10 Hz over a 365micron laser fibre used to ablate the rest of the remaining visible tumour in the distal left ureter. The left nephrostomy was removed following successful placement of a retrogradely placed left JJ stent.

## DISCUSSION

The management of completely transected or obliterated ureteral injuries is complex and poses several medical challenges with patients requiring multiple surgical or radiological procedures. Most often, a passage of a ureteric stent across the affected segment fails and ultimately patients may require urinary reconstruction including ureteric re-implantation, with vesico-psoas hitch or even Boari flap. These procedures traditionally performed with an open technique are associated with increased morbidity and longer times to recovery, although laparoscopic and robotic assisted techniques may be associated with advantages such as lower blood loss, better cosmetic results and quicker recovery times [3, 4].

A rendezvous technique can be used to restore the continuity of the urinary tract and most importantly render a patient nephrostomy-free. ‘Success’ of a combined antegrade/retrograde procedure is dependent on many factors including stricture characteristics and requires a multi-disciplinary approach to select appropriate patients. We envisage our technique to be useful in patients with iatrogenic obliterated intramural ureter. Long-term follow-up with our patient from Case 1 shows good functional recovery at 3 years without requirement of a ureteral stent.

The rendezvous procedure to cross-complicated ureteric strictures has been minimally described in the medical literature, however, none have described techniques used to manage completely obliterated ureteric orifices. Watson *et al.* described a technique to manage ureteric strictures with a success rate of 100% in 20 patients with only three patients requiring further surgical intervention (one nephrectomy, two urinary tract reconstructions) within 3 years of follow-up [5]. Pastore *et al*. described a case series of 18 patients following iatrogenic ureteric injury following gynaecological surgery. Similarly, reported restoration of ureteral continuity was 66% (12/18) [6].

Our described approach provides a unique way to manage complete ureteric orifices occlusion. Based on our experience, surgeons with subspeciality experience in endourology should be in a position to manage a complete ureteric obstruction via this approach. It is crucial to have a thorough understanding of the complications which can occur such as bladder perforation, and significant ureteric injury leading to functional nephron loss. Correct radiological positioning of the Collins knife above the antegrade guidewire is imperative to reduce the extension of the bladder perforation and to allow accurate access at the ureteric cul-de-sac.

The two cases of our series have different characteristics, but similarly good outcome. The first case was a recent bilateral ureteric obliteration at the level of the vesico-urethral anastomosis, whereas the second one was an intramural ureteric obliteration due to scar tissue formed by previous TURBT. On this second case the neo-access allowed a successful ureteroscopy and laser ablation of ureteric tumour. On both cases the approach was identical and uneventful. This indicates that our technique is reproducible and standardized. Balloon dilatation might be required if there is an excess of surrounding scar tissue, or if the neo-ureteric orifice appears to be tight around the guidewire.

Throughout our experience, the first patient with bilateral ureteric obstruction suffered a fungal urosepsis after his endoscopic repair, possibly due to inadequate sterilization of his nephrostomy sites. He was managed successfully with oral Fluconazole, which had to be continued until the date of his stent removal. This episode could have contributed to the drop of his baseline eGFR from readings of >90 ml/min to readings between 50 and 60 ml/min. However, his latest eGFR improved to 66 ml/min, 3 years from his repair, which indicates satisfactory and long-lasting functional outcome. On the occasion of the second case, his antegrade manipulation was undertaken after his nephrostomy had been prepped with Betadine Iodine solution and was uncomplicated.

## CONCLUSION

Ureteric injury is a rare but well-recognized complication of urological surgery. To our knowledge, this is the first report to set out two cases of iatrogenic complete ureteric obstruction managed successfully with an endoscopic approach to restore continuity of the ureter. Both patients avoided major reconstructive procedures. These cases prove that our described endoscopic approach is an alternative method to well-recognized approaches of open or laparoscopic ureteric re-implantation and offers excellent long-lasting functional outcome.

## AUTHORS’ CONTRIBUTION

S.B and M.K researched the surrounding literature and were involved in the writing of the article. S.B and M.K gained informed consent (written). S.B, M.K and A.A made appropriate changes to the draft article. All authors reviewed and approved the final article prior to submission.

## References

[1] Koc E, Canda A. Robotic urologic surgery complications. Mini-invasive Surg 2018;2:7.

[2] Chalya P, Massinde A, Kihunrwa A, Simbila S. Iatrogenic ureteric injuries following abdomino-pelvic operations: a 10-year tertiary care hospital experience in Tanzania. World J Emerg Surg 2015;10:17. 25774212 10.1186/s13017-015-0011-z PMC4359460

[3] Gild P, Kluth L, Vetterlein M, Engel O, Chun F, Fisch M. Adult iatrogenic ureteral injury and stricture–incidence and treatment strategies. Asian J Urol 2018;5:101–6. 29736372 10.1016/j.ajur.2018.02.003 PMC5934506

[4] Burks F, Santucci R. Management of iatrogenic ureteral injury. Ther Adv Urol 2014;6:115–24. 24883109 10.1177/1756287214526767 PMC4003841

[5] Watson JM, Dawkins GPC, Whitfield HN, Philp T, Kellett MJ. The rendezvous procedure to cross complicated ureteric strictures. BJU Int 2002;89:317–9. 11856118 10.1046/j.1464-4096.2001.00587.x

[6] Pastore AL, Palleschi G, Silvestri L, Leto A, Autieri D, Ripoli A, et al. Endoscopic rendezvous procedure for ureteral iatrogenic detachment: report of a case series with long-term outcomes. J Endourol 2015;29:415–20. 25226409 10.1089/end.2014.0474

